# N^3^-MEA Probes: Scooping Neuronal Networks

**DOI:** 10.3389/fnins.2019.00320

**Published:** 2019-04-10

**Authors:** Dmitry Kireev, Viviana Rincón Montes, Jelena Stevanovic, Kagithiri Srikantharajah, Andreas Offenhäusser

**Affiliations:** ^1^Forschungszentrum Jülich, Institute of Bioelectronics (ICS-8), Jülich, Germany; ^2^Department of Electrical and Computer Engineering, University of Texas at Austin, Austin, TX, United States

**Keywords:** microelectrode array, neuronal networks, neural probes, advanced neurotechologies, brain-computer interface

## Abstract

In the current work, we introduce a brand new line of versatile, flexible, and multifunctional MEA probes, the so-called *Nano Neuro Net*, or N^3^-MEAs. Material choice, dimensions, and room for further upgrade, were carefully considered when designing such probes in order to cover the widest application range possible. Proof of the operation principle of these novel probes is shown in the manuscript via the recording of extracellular signals, such as action potentials and local field potentials from cardiac cells and retinal ganglion cells of the heart tissue and eye respectively. Reasonably large signal to noise ratio (SNR) combined with effortless operation of the devices, mechanical and chemical stability, multifunctionality provide, in our opinion, an unprecedented blend. We show successful recordings of (1) action potentials from heart tissue with a SNR up to 13.2; (2) spontaneous activity of retinal ganglion cells with a SNR up to 12.8; and (3) local field potentials with an ERG-like waveform, as well as spiking responses of the retina to light stimulation. The results reveal not only the multi-functionality of these N^3^-MEAs, but high quality recordings of electrogenic tissues.

## Introduction

The current state-of-the-art in neural technology has advanced in numerous and different approaches. A vast variety of devices have been designed and proposed in the last decades, creating unique tools to address specific tasks, such as penetrating and surface electrodes for the brain (EEG, ECoGs, intracortical implants) (Ghane-Motlagh and Sawan, 2013; Scholten and Meng, 2015), the retina (epiretinal and subretinal implants) (Lewis et al., 2016), or the cochlea (cochlear implants) (Clark, 2015), and sieve or cuff electrodes to address individual nerves from the central (optic nerve) and peripheral nervous system (Stieglitz et al., 1997; Khodagholy et al., 2015; Weltman et al., 2016; Birenbaum et al., 2017). Nonetheless, these probes are typically unifunctional, each specifically shaped into one specific application. As an example, micro-electrocorticography (μECoG) application and μECoG devices are one of the most commonly fabricated and used to study brain due to its mild invasiveness and high signal fidelity (Castagnola et al., 2015; Khodagholy et al., 2015; Insanally et al., 2016; Kaiju et al., 2017). Density of electrodes in the μECoG array may define the final application, and can range from treating diseases to building prosthetic tools and advanced brain-machine-interfaces (BMI) (Stieglitz et al., 2009). The state of technology in the μECoG has advanced toward ultra flexible (Khodagholy et al., 2015) and even current bioresorbable (Yu et al., 2016) devices capable of recording signals with fidelity and resolution (Kim et al., 2010; Kozai et al., 2012).

The contemporary upgrades in the design of neural probes have started with the fabrication of soft and flexible probes, as a way to develop compliant and tissue-like devices. In order to reduce the mechanical and biological mismatch between the neural devices and its target, different polymers such as polyimide, SU-8, parylene-C, or silicone rubber have been used as substrate materials (Margolis and Detwiler, 2007; Lacour et al., 2016). Furthermore, in order to reduce the impedance and maximize the charge injection capacity of the electrodes, the use of simple gold has moved toward new materials, such as Pt black, PEDOT:PSS, graphene, etc. (Fattahi et al., 2014; Massobrio et al., 2016; Kireev et al., 2017b; Liang et al., 2018). Nonetheless, regarding electrode material development, there is an alternative path, and the path has been confirmed by a number of thorough studies unveiling that a simple gold interface is good enough for the most modern *in vivo* and *ex vivo* applications. A lot depends not directly on the electrode and the respective interface with the tissue, but the tissue itself, the mechanical and biological properties of the device, hydrophobicity, etc. (Neto et al., 2018).

In biomedical research, microelectrode arrays (MEAs) are used as tools to interface electrogenic organs *in vivo*, acute tissue slices *ex vivo*, or 2D and 3D cell cultures, in order to measure and even stimulate the cells to establish a BMI connection or treat diseases. When approaching long-term cell cultures on planar MEA chips, the rigid substrates of standard MEAs affect cell adherence, proliferation, shape, and growth when compared to *in vivo* cells, as physiological and mechanical aspects in the environment are not the same (Kapałczynska et al., 2018). In this way, extrapolating the findings from these planar recordings into *in vivo* models is rather inappropriate. Several approaches have been proposed to overcome this obstacle. Different kinds of mesh electronic and ultraflexible penetrating probes were developed mainly with the purpose of *in vivo* recordings from cortex (Hong et al., 2018; Wei et al., 2018). However, application of these devices is somehow limited and highly specified, usually requiring complicated surgical operations of implantation and measurement of localized area of cells.

**Figure 1:**
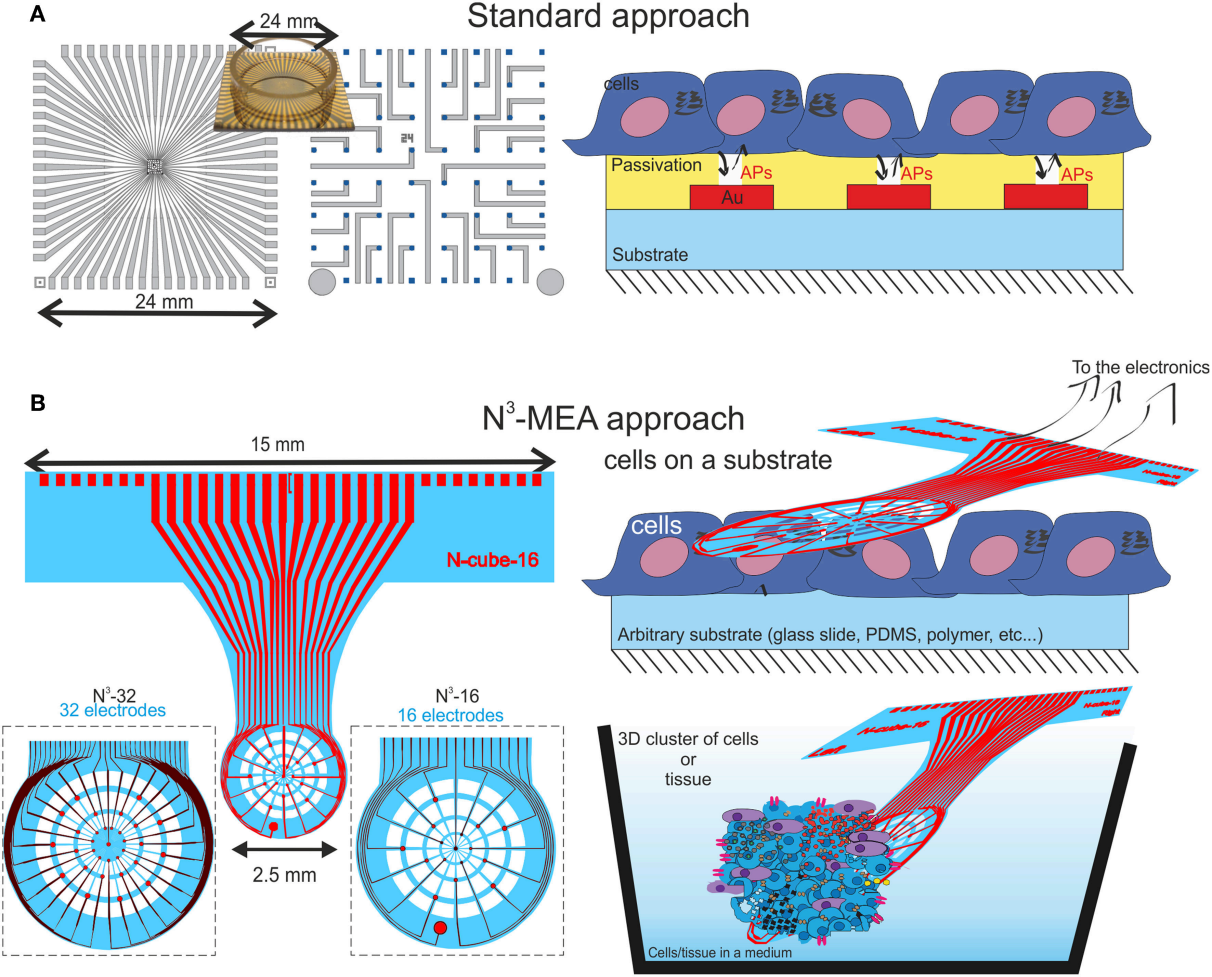
A schematic comparison between a classical approach for cell electrophysiology **(A)**, where the chips are most commonly rigid and the cell are grown on top of a typically flat and rigid chip. In **(B)** is shown the schematics of the proposed N^3^-MEA probes that are flexible yet robust, semi-transparent and holey, allowing cells to grow through the probe or the probe to be placed on top of the pre-grown cell in order to increase the throughput of electrophysiological measurements.

Multi-functionality is another, commonly overlooked feature that is essential for the future development of the field: instead of spending tremendous efforts to create precision tools for rough experiments, it might be better to have a probe that can fit to a variety of tasks and applications. What is a common problem for scientific groups is to advance their research from *in vitro* studies to further real *in vivo* measurements. And the problems are technological. Most of the *in vitro* based devices are planar (see Figure 1A). Even when fabricated on a flexible substrate, such devices are typically planar, and in some way, fixed in order to culture cells on top of it (see Figure 1A for a schematic overview). The main scientific doubt here is following: can the findings recorded on such platforms be extrapolated to the *ex vivo* and *in vivo* models (Kapałczynska et al., 2018)?

In this work we propose a novel tool, *Nano Neuro Net* (N^3^)-probe, that can solve the problem and play a role of a transient tool between *in vitro* and *in vivo* recordings. As shown in Figure 1B, the probe itself is ultrathin and flexible, and is manipulated separately, while cells can be cultured on arbitrary substrates, or even whole tissue slices can be used as test subjects. When the time is right, when the cells are mature enough to produce electrical signals the probe can be brought into contact with the cell culture for electrophysiological measurements. This is a great advantage of the N^3^-MEAs compared to the common planar MEAs: the latter are used to grow the cell on top of them, which might take days, weeks, or even month of time in incubator. In the case of N^3^-MEAs, however, it is possible to use a single probe, and a number of different cell lines, tissues, that are cultured separately, and make multiple recording with one device for more valid comparative measurements.

The N^3^-probes reported in the work are shown to successfully record action potentials from heart tissue cells, spontaneous activity of retinal ganglion cells, and local field potentials with an ERG-like waveform as a proof of principle. Yet, we believe the N^3^-probes have potential to cover a much larger set of application, including chronic applications. The proposed N^3^-probes are multifunctional and can further be upgraded toward measurements from *in vitro* cell culture to *ex vivo* recordings of electrogenic tissues and even more complex 3D cultures of neurons or any other cell type. Moreover, the shape, dimensions, and functionality of the N^3^-probes allow to think beyond typical neuronal recordings toward the application of neural prostheses, such as μECoG on-dura brain tissue or retinal implants. An important remark on the fabrication of such tool has to be made, as our fabrication routine does not require e-beam lithography [as opposed to reference (Luan et al., 2017) for an example], but the whole process is fully based on UV photolithography, upscaling drastically the throughput of the method. In this way, the use of these probes will also promise higher throughput of the recordings, as in this case the researchers are not limited by the number of probes and cell culture time, while, it can be possible to use even just one probe/device for as many recordings (from different cultures) per day as possible.

## Methods

### N^3^-MEA Fabrication

The N^3^-MEAs are fabricated via UV photolithography on 4-inch wafers in order to upscale the fabrication throughput and reduce the costs. The fabrication begins with e-beam assisted evaporation of Cr/Au/Cr layer on top of the bare Si wafers. This triple layer serves as a sacrificial layer and is etched away in Cr etchant as the last step. The first layer of polyimide is formed via spin-coating of PI-2611 (HD Microchemicals) on the wafers with the rate of 5,000 rpm, that is later soft baked at 120°C for 4 min (with a gradual ramp up of the temperature) and hard baked in a convention oven filled with nitrogen atmosphere at 350°C (the temperature is ramped up to 200°C with rate of 4° C/min, held for 30 min, then raised up to 350°C with rate of 2.5°C/min, held for 30 min and then slowly cooled down). Such process ensures a smooth and pinhole free polyimide layer of ~2.5 μm in thickness. Further, a layer of Ti/Au (10/200 nm) is formed on top via an e-beam assisted evaporation and is shaped into the desired structure by means of lift-off process through a double layer of nLOF-2020 (HD Microchemicals) and LOR-3B photoresists (Microchem). A second layer of polyimide is then spin-coated, soft-baked and hard baked in exactly the same manner as explained above with only additional step of spin-coating the adhesion promoter, VM-652 (HD Microsystems), beforehand in order to enhance the interlayer adhesion. Later, two reactive ion etching (RIE) steps through two different masks were performed in order to etch away: (i) the electrode openings (only top 2.5 μm PI), (ii) the desired shape of the probes (~5 μm of PI). Both steps are performed by using AZ-9260 as a thick protective photoresist and O_2_/CF_4_ as gas combination to etch the polyimide. The AZ-9260, spin-coated at 3,000 rpm results in approximately 9 μm thick layer, that is thick enough to ensure complete etching of the 5 μm PI layer. The RIE etch was performed in the mixture of O_2_/CF_4_ (36/4 sccm) gases with RF power of 50W, ICP power of 500 W, and pressure of 0.007 Pa. As we determined experimentally, these conditions result in the etch rate of the most polymers, such as polyimide, parylene-C and most of the common photoresists of 700–800 nm/min. Therefore, the etching times of 4 and 8 min were used to etch the passivation openings and shapes, correspondingly. The wafer is further diced into smaller clusters in order to simplify the Cr etching and probe release process. See Figure S1 for detailed schematic of the fabrication flow.

When the fabrication is finished, the chips are immersed into Cr etchant (Sigma Aldrich) for 2 h. Typical etch rate of Cr/Au/Cr layer with only sidewall access was found to happen at approximate rate of 1.5 mm/hour. When the etching is finished, the probes are washed thoroughly in water, and dried with N_2_ flow. Important to note that the PI-based probes of only 5 μm are not just stable enough to sustain the process, but can be hardly broken if specifically intended to (see Supplementary Movie 1).

**Figure 2:**
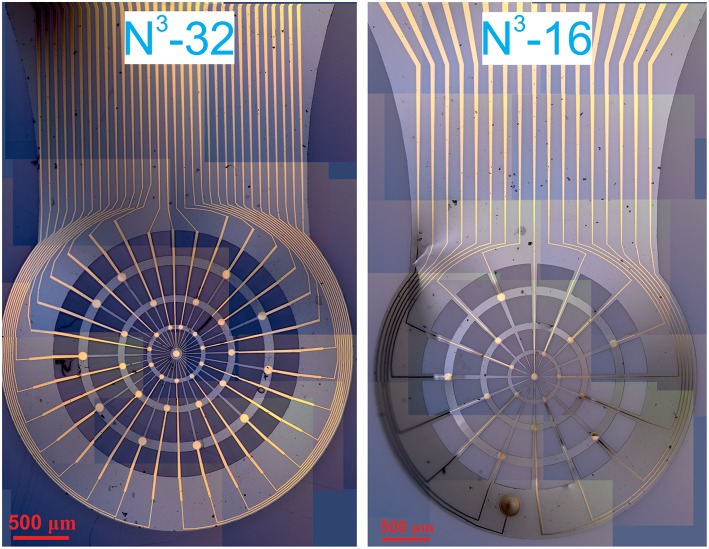
Panoramic assemblies of optical pictures of the N^3^-32 probe(left) and N^3^-16 probe (right).

The fabricated probes have dimensions of 15 mm in width and 3 mm in length of the back-side (that is later connected to a PCB carrier), while the active part is only ~2.5 mm in diameter, where the electrodes are located radially on the nodes. The diameter of the electrodes as well as their distribution is selected to fit the wide range of applications and varies from 30 to 80 μm. Details of the design can be found in the Figures S2, S3. The optical images of the active areas of the N^3^-probes are given in Figure 2 as well as they higher quality images are available in Figures S4, S5.

### Impedance Spectroscopy

Electrical impedance spectroscopy study is performed on VSP-300 (BioLogic Science Instruments) potentiostat. The measurements are performed in a simplified three electrode schematics with Ag/AgCl pellet used as both reference and counter electrode since there is no expected current flow in the system. The spectra are taken in a frequency range from 1 Hz to 100 kHz in 1x PBS solution, with 10 mV AC potential applied.

### Electrophysiology

The extracellular recordings are performed with the in-house developed biological multifunctional amplifier system (BioMAS), which is capable of recording up to 64 channels simultaneously with a sampling rate of 10 kHz per channel, as well as allows a maximum signal amplification of 1010 (10.1 pre-amplification and either 1, 10, or 100 in a second amplification phase) (Krause et al., 2000; Kireev et al., 2017a). Additionally, a 3 kHz anti-aliasing low pass filter is installed in the amplifier system. Typically a Ag/AgCl pellet electrode (Warner Instruments) is used as a reference. The process is controlled via LabView based software. Supplementary Movie 2 shows the above-mentioned system, set-up and probe handling.

### Probe Assembly for *ex vivo* Measurements

The BioMAS system is a multi-unit system that, however initially was built for *in vitro* cell culture recordings. In order to establish a seamless integration of the flexible probes with the standard BioMAS system and enable the *ex vivo* recording with the flexible N^3^-MEAs, we modified the pre-amplifier specifically to have rather long connectors (ca. 30 cm) ending with a set of pins. The long connectors do possibly introduce more noise into the recordings, yet as will be shown later, the measurements are robust enough to not be significantly affected by the noise.

The probes, in their turn, are flip-chip bonded to a custom made printed circuit board (PCB) carrier of approximately 10 cm long and 15 mm wide where the inner pads match the contact pads design of the N^3^-probes. The carrier is placed on a hotplate (180°C), and a solder paste (42Sn/58Bi alloy, NC-31, AMTECH) is dispensed on top of the contact pads. When the flux is evaporated and the excess alloy is removed, only small amounts of alloy is left on top of the carrier's contact pads. Then, the N^3^-probe is placed on top of the carrier and aligned under the microscope. When cooled down, any remaining flux is removed in ethanol, and the probe is carefully glued with medical grade epoxy (EPO-TEK 302-3M). Optical images of the N^3^-probes as well as zoom-in into the PCB-probe assembly is given in Figure S6.

After each electrophysiological measurement, the N^3^-probes were thoroughly washed with DI water followed by a 1-h soak in Terg-a-zyme solution in order to eliminate any organic residue and washed with DI water again.

### Heart Tissue Preparation

The heart tissue is prepared by dissecting embryonic tissue from 18 weeks pregnant Wistar rat. Pregnant rats were anesthetized with CO_2_ and decapitated. The pups were then removed and also decapitated under sterile conditions. The heart of an embryo was quickly isolated, washed in Hank's balanced salt solution (HBSS), then stored and measured in supplemented Claycomb medium. More details of the heart tissue preparation can be found elsewhere (Hersch et al., 2013). The experiments are done with the approval of the Landesumweltamt für Natur, Umwelt, und Verbraucherschutz Nordrhein-Westfalen, Recklinghausen, Germany, number 84-02.04.2015.A173.

### Retina Preparation

Light-adapted retinas were isolated from a 3 months old C57BL/6 wildtype mouse (Charles River). The animal was anesthetized with isoflurane (Deltaselect, Actacis Dtl. GmbH&Co. KG) and killed by decapitation. This procedure was carried out in accordance to the German Law for the Protection of Animals and after approval was obtained by the regulatory authorities, the Forschungszentrum Jülich, and the Landesamt für Natur, Umwelt und Verbraucherschutz of the land North-Rhine Westfalia. The eyeballs were then enucleated and perfused with oxygenated Ames' medium (Sigma-Aldrich) at a pH of 7.4 at room temperature. The cornea, the lens, and the vitreous body were first removed for each eye, followed by the isolation of the retina from the eyecup. The tissue was then cut into halves and each piece was mounted on top of a millipore filter paper (Merck KGaA, Germany) with a precut hole of 1.5 mm in the middle. In this way, the filter paper was used as a carrier to transfer the retina onto the device, and the precut hole allowed the retina, with the ganglion cell layer (GCL) facing downwards, to contact the electrodes. Furthermore, a glass ring glued the lid of a petri dish was used to create a reservoir of the physiological solution, and a silicone rubber pillow served as support for the N^3^-probe and the retina.

During the experiments, carbogen gas (The Linde Group, Germany) was used to constantly oxygenate the medium, and a perfusion system was used to maintain the tissue vital with fresh medium. In order to observe physiological responses of the retina, optical stimulation was carried out with a light-emitting diode (LED) using 500 ms light pulses every 20 s. In addition, to confirm the responses of the retina evoked by light, the electrical activity of the tissue in a hypoxic/ischemic state was performed after stopping the perfusion of fresh oxygenated medium to the tissue. At last, to confirm the spiking activity of RGCs, the electrical activity was recorded when the tissue was on top of the electrodes and when the tissue was being removed.

## Results and Discussion

As it is shown in the methods section above, our devices feature two kinds of probes, with either 16 or 32 electrodes, namely N^3^-16 and N^3^-32. In both of the probes, the electrodes are arranged radially through the web-like net of polyimide that is made specifically to be strong and stable in all directions. However, the N^3^-16 probes feature electrodes with openings of 30, 50, and 70 μm in diameter, and one on-probe reference electrode of 200 μm diameter (see Figure 2 and Figures S2, S4). The N^3^-32 probes feature electrodes with diameters of 40, 60, and 80 μm (see Figures S3, S5). Importantly, in order to mimic the distribution of neurons in retina, the density of the electrodes is higher in the center of the probes, while the diameter of the electrodes is lower. On the outskirts of the probes, however, the electrodes are larger yet sparser in distribution.

**Figure 3:**
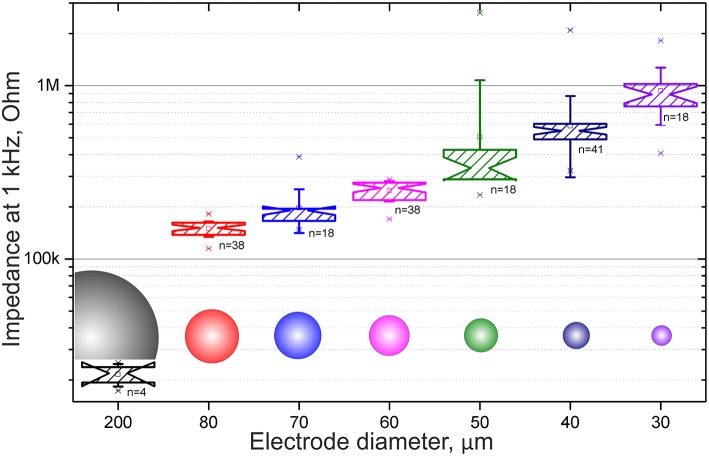
Statistical data plots of the electrode impedance at 1 kHz depending on the diameter of the electrodes.

The main parameter as typically taken into account as a figure of merit when talking about MEAs and extracellular measurements is the impedance of the electrodes, i.e., property and quality of the metal layer that is essentially interfacing with the environment. Figure 3 shows the statistical distribution of the impedance of the electrodes at 1 kHz depending on their diameter. The impedance of our electrodes is that of typical gold-based electrodes, and ranges from 930 ± 340 kOhm for the smallest electrodes (30 μm in diameter) to 150 ± 15 kOhm for the largest electrodes (80 μm in diameter).

**Figure 4:**
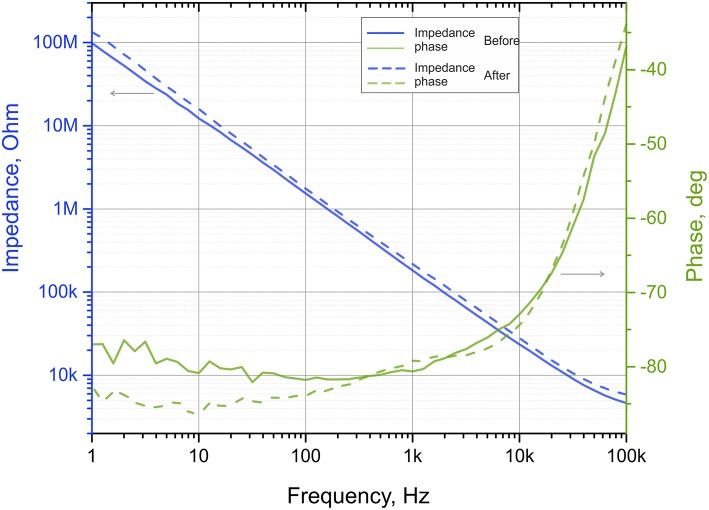
Impedance plots of the same electrode as fabricated (before), and after a series of electrophysiological measurements. Solid lines represent impedance and phase shift before while dashed lines represent data from the used device. The electrode is 80 μm in diameter and is #13 in the Table S1.

Important to note, the N^3^-probes are designed and tested to be reusable and we have used for extracellular recordings multiple times. In order to check stability of the probes, we measured impedance of the devices before as well as after the cell culture and tissue electrophysiology to make sure that no degradation of the electrodes or damage of the feedlines happen during the measurements. The representative Bode plot of the impedance of the 80 μm diameter electrode is shown in Figure 4. We found that in average >94% of the devices survive the measurements, yet the impedance of the electrodes increased slightly, by the 32 ± 10% (see Table S1 for details). As one can see, changes of impedance before and after several experiments are minor, allowing us to reuse the N^3^-probes multiple times.

The recordings from *ex vivo* heart tissue, *in vitro* cultures of HL-1 cells (see Supplementary Information for details), and *ex vivo* retinal ganglion cells shown later in the manuscripts are result of multiple experiments performed on not just freshly made, but re-used N^3^-probes. Cleaning of the N^3^-probes was performed in terg-a-zyme solution (see methods).

### Heart Tissue Measurements

In order to show the applicability of the N^3^-probes toward multifunctional electrophysiology, we started with possibly the most robust *ex vivo* electrogenic tissue: heart tissue. Prepared as explained in the methods section of the manuscript, the embryonic heart tissue is kept floating in the supplemented Claycomb solution.

**Figure 5:**
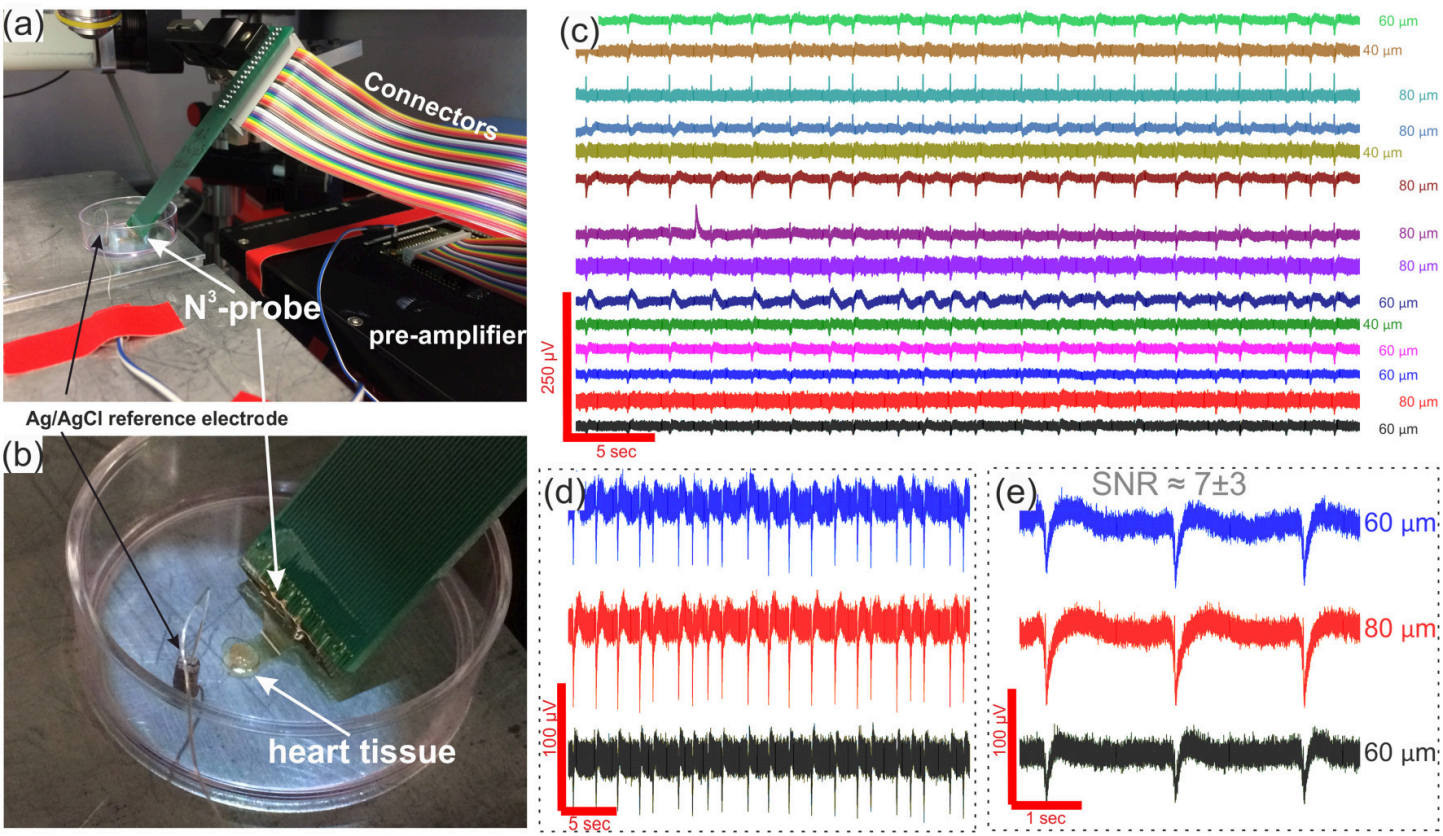
Heart tissue electrophysiological recordings. In **(a,b)**, optical images of the set-up consisting of a pre-amplifier, connectors, reference electrode, and N^3^-probe that is placed directly on top of the embryonic heart tissue. **(c)** Timetrace recordings from 14 electrodes with signal to noise ratio between ~ 4 and ~ 10; **(d)** and **(e)** and zoom-ins into three of the representative timetraces to show the shape of the APs as well as absence of spatial signal propagation in the recordings. Corresponding electrode diameters are shown to the right of each timetrace.

The N^3^-probe, bonded to a PCB carrier and connected through a wire cord to the pre-amplifier (see Figure 5a). The distance between probe and pre-amplifier must be as little as possible in order to eliminate any interference before signal is pre-amplified (x10). The probe is fixed via a custom made manipulator in order to allow sufficient range of x-, y-, and z- plane movement of the probes for manually moving the probe for scooping the electrogenic tissue. A Ag/AgCl pellet electrode is used as a ground and reference electrode is wired up separately (see Figures 5a,b for details).

While performing the *ex vivo* measurements, we realized that the contact/adhesion between the tissue and the N^3^-probe immersed in electrolyte is not ideal. We believe that the reason behind the poor adhesion is mechanical mismatch and flexural stiffness of the probe and believe this can be solved by further decreasing the thickness of the probes, consequently improving the probe-tissue conformability (Kim et al., 2010; Lacour et al., 2016). The importance of reduced thickness is associated with the flexural stiffness of the material, that is proportional to the cube of probe's thickness (Lacour et al., 2016). Therefore, the thinner the material the more bendable it is. This is critically important in order to establish a tight contact with the tissue. As studied before, PI mesh of thickness 2.5 μm provides excellent mechanical contact and wrapping of the tissue (Kim et al., 2010). Another possible way to improve the sealing between the probe and tissue/cells is to prolong the contact time toward hours (rather than minutes as in our experiments). This will, however require multiple changes to the set-up, as it will require in-incubator recordings (yet can be done, see proposed schematic in Figure S11). Nonetheless, in order to perform the heart tissue recordings, once the N^3^-probe is carefully and slowly placed directly on top of the heart tissue, we remove most of the supplemented medium in order to ensure a firm contact between the tissue and probe. There are, moreover, several other means that might possible be used instead or in combination with the proposed method to even further improve the probe-to-tissue contact, e.g., oxygen plasma treatment or pre-measurement poly-L-lysine coating of the N^3^-probe. Both of the methods would increase hydrophilicity of the polyimide, resulting in a more natural and tight contact with the tissue. Nevertheless, the level of liquid left is enough to still ensure contact with the tissue and grounded through the Ag/AgCl pellet reference electrode. Once the tight contact between the N^3^-probe and heart tissue is established, it is possible to record the extracellular signals that are shown in Figures 5c–e. In this exact case we were able to pick up the signals from 22 electrodes with an average AP amplitude of 38.52 ± 3.72 μV, a maximum peak amplitude of 99 μV, an average signal to noise ratio (SNR) between 4.22 ± 0.37 and 9.87 ± 1.39, and a maximum SNR of 13.2 (see Figures 5d,e). While the AP amplitude seem to be rather poor, we believe this can be explained by an imperfect adherence between the N^3^-probe and tissue and consecutively a large gap and large leakage of the signal. As it can be seen better from a zoom in into APs on adjacent electrodes, there is no visible signal propagation through the recordings, which is an evidence of the classic heart tissue that produces the signal synchronously through the whole organ.

### Retina Measurements

**Figure 6:**
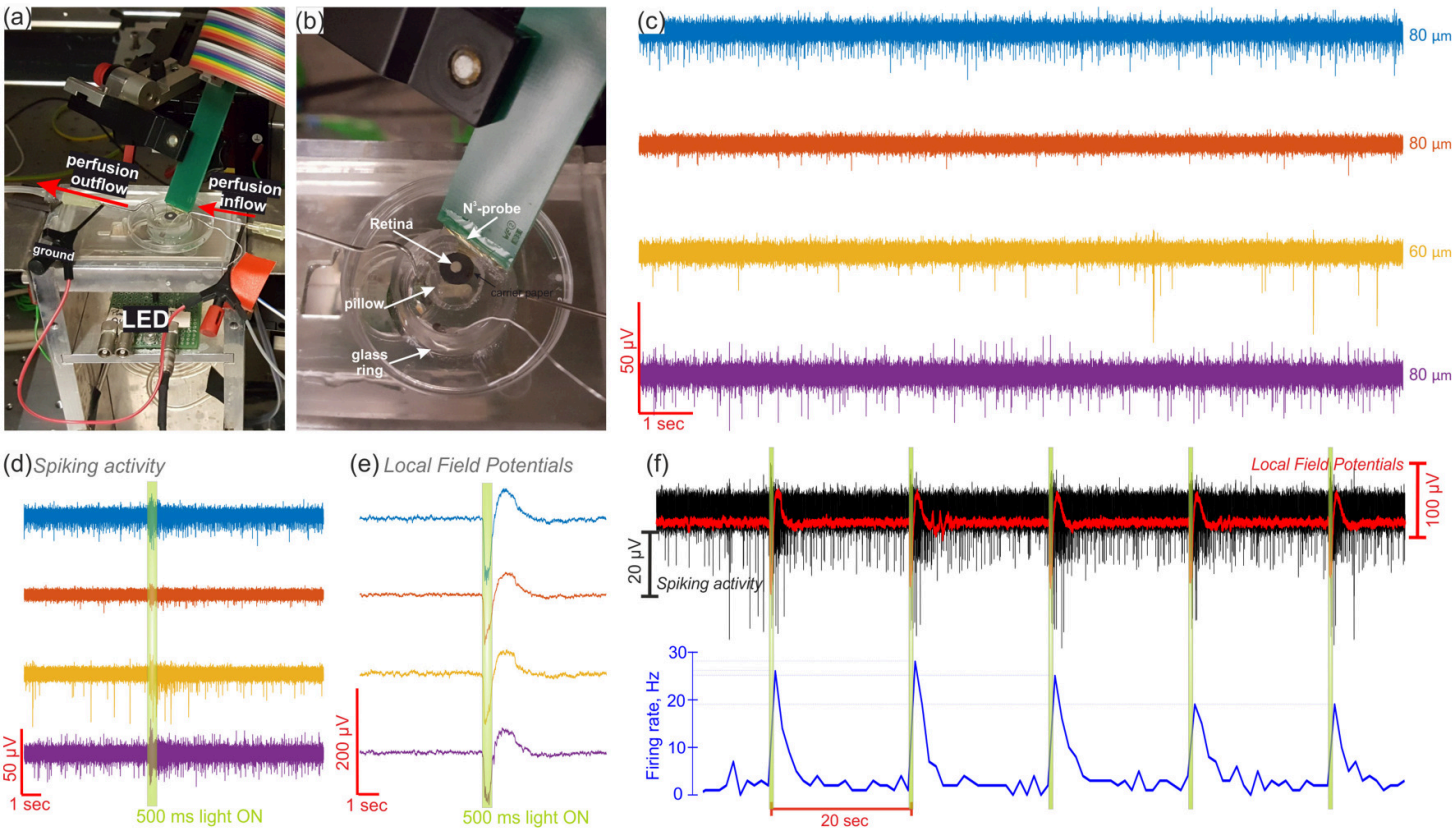
Electrophysiological recordings of the retina. In **(a)** and **(b)** are photos of the experimental setup used to record from the retina. The N^3^-probe was used in an upside up mode, as the tissue was placed on top of the device, and a light-emitting diode (LED) was used from beneath to perform optical stimulation of the tissue. In **(c)**, the spontaneous activity of retinal ganglion cells (RGCs) captured in four different channels and the respective electrode diameters shown to the right. A 500 ms light pulse, depicted as a green highlight in **(d–f)**, was used to stimulate the retina, eliciting both, spiking **(d)** and low frequency signal **(e)** responses. The electrical activity in **(c–e)** are color-coded and correspond to the same four channels. In **(f)**, the spiking activity (in black), the LFPs (in red), and the firing rate (in blue) are shown along 130 s during repetitive light stimulation with 500 ms light pulses every 20 s (green highlights). The electrical activity shown in **(f)** corresponds to the same yellow recording channel in **(c–e)**.

To prove the capability of the N^3^ probes to approach neuronal tissue, they were tested with explanted retinas. Using the device in an upside mode, the isolated retina was transferred on top of the device with the GCL facing the electrodes. Once the tissue was in contact with the electrodes, Ames' medium was poured inside the glass ring. A reservoir with constant inflow and outflow of the oxygenated physiological solution was then generated to keep the tissue vital during the experiment. In this way, the medium was covering completely the tissue-electrode interface and the Ag/AgCl reference electrode used for the retinal recordings (see Figures 6a,b).

The N^3^-probes have shown to successfully record the neural activity of the retina (see Figure 6c). While the electrodes were in contact with the GCL, the spiking activity of different retinal ganglion cells (RGCs) was captured, as exhibited by four recording channels in Figure 6d (see more recordings from another retina in Figure S7). Here, when no stimulus was present, the typical spontaneous and maintained activity of RGCs with continuous and stochastic spikes (Margolis and Detwiler, 2007; Nelson, 2007) was recorded by different electrodes. Furthermore, it was possible to capture physiological responses to optical stimulation using a 500 ms light pulse every 20 seconds. Consequently, an increased number of APs was distinguished when the optical stimulus was ON, revealing ON sustained responses in the spiking activity of the RGCs (Awatramani and Slaughter, 2000; Nelson, 2007). This effect was confirmed when analyzing the spike count along a complete recording. For example, Figure 6f shows the complete recording of the yellow channel exhibited in Figures 6c–e, in which the firing rate increased in average from 2.2 to 23.4 Hz after the ON switch of every light stimulus.

In order to derive the source of the spiking activity captured by the recording electrodes, the waveforms of the APs were extracted. In this way, waveforms with durations between 1.5 and 2 ms and negative peaks, comparable to the biphasic shape of retinal somatic spikes, as well as spikes with an initial positive crest followed by a negative peak, akin to the triphasic appearance of axonal APs (Segev et al., 2004), were observed among the different electrodes (see Figure S8). Likewise, a follow-up of the amplitude and the outline of the APs detected by the electrodes before, during, and after optical stimulation, exposed the presence of more than one spiking unit per electrode (see Figure 6d, S8).

Moreover, local field potentials (LFPs) exposing an electroretinogram (ERG)-like waveform were captured in response to light stimulation (see Figures 6d,e). Such potentials displayed a negative wave followed by a positive peak, which are comparable, respectively to the a- and b-waves of the ERG of light-adapted mouse retinas. While the literature suggests a low amplitude a-wave (~ −50 μV) and a big amplitude b-wave (~ −200 μV) for light-adapted mouse retinas (Levin and Adler, 2011), greater a-waves and diminished b-waves were captured, therefore implying a poor electrode-tissue coupling (Fujii et al., 2016). Accordingly, a weak contact between the neural tissue and the device could explain the low spikes amplitudes captured in the recordings, which presented a maximum negative amplitude of 42.3 μV. However, a low background noise, which ranged between 4.9 and 6.8 μV, enabled high SNRs between 4.7 ± 0.9 and 5.6 ± 0.7 and a maximum SNR of 12.8.

Furthermore, in order to confirm that the spikes and LFPs elicited during light stimulation were not artifacts induced by light but a physiological response of the retina to light, the same explanted retina was stimulated with the same light stimuli after 1 h and 20 min without fresh oxygenated medium. During the hypoxic/ischemic state of the retina, most electrical activity was gone, however spontaneous APs were still captured from the retina in one channel. In addition, some low frequency waves were seen during light stimulation, however these LFPs were low in amplitude (~ ±10 μV) and in the range of the background noise. Therefore, neither visible spiking reactions, nor ERG-like signal responses to the stimuli were captured (see Figure S9). These results agree with the literature, in which a reduced spontaneous activity of RGCs can still be captured during hypoxia (Alder and Constable, 1981) and ERG signals are diminished and lost in rodents when the perfusion of the retina was stopped (Block and Schwarz, 1998; Jehle et al., 2008). The latter confirms then that the spiking and ERG-like signals responses observed in the perfused retina after optical stimulation were evoked by the light stimuli. Additionally, the retinal activity recorded during the experiments was confirmed to be product of the electrical activity of RGCs, as the spiking activity captured by the electrodes was lost once the tissue was removed from the electrodes and the background noise was reduced to ~ 4 μV, since the electrical resistance of the tissue was not present anymore (see Figure S10).

## Conclusions and Outlook

The novel multifunctional, ultrathin, robust and versatile N^3^-MEA probes that are introduced in this work are shown to be advantageous for manifold electrophysiological measurements. The unique physical structure of the N^3^-MEA allows a seamless integration with neuronal cells and tissues, resulting in low noise and large SNR recordings. We have shown ability to record electrical signal from heart tissue cells with SNR up to 13.2 as well as from retinal ganglion cells with SNR up to 12.8.

We believe that these N^3^-probes can play the role of a transient tool between *in vitro* and *in vivo* recordings. The application range is not limited to the two applications reported in the manuscript, i.e., recordings from heart tissue and retinal tissue. In contrary, we believe that the proposed design, shape, dimensions, and functionality of the N^3^-probes allow to go beyond such neuronal recordings toward the application of neural prostheses as μECoG on-dura brain tissue or retinal implants. In order to perform the chronic ECoG measurements, or to measure electrical activity of 3D neuronal cell culture, the PCB connector board can be simply substituted with a flat flexible cable (see proposed schematic in Figure S11). Further advancing can be done via reducing probe's thickness down to 1–2 micrometers. Technologically, it should also be possible to fabricate the devices on much thinner polyimide and parylene-C dielectric polymers. Going into even thinner layers of polyimide would allow a much better and seamless integration with tissue. This reduction in thickness will result in improved flexural properties of the N^3^-probes, therefore improving the contact with tissue. Second advance of the probes we envision to come from utilizing other materials instead of simple gold, such as CNT, Pt black, PEDOT:PSS, porous graphene or single layer graphene. Moreover, usage of graphene, in our opinion, will bring the third advance to the future work: intrinsic flexibility, conductivity and transparency opens interesting prospects for optogenetics and retinal applications. Thinning down the probe thickness as well as use of graphene or other two-dimensional materials will bring us closer to the term *nano* claimed in the name of the N^3^-probes. Stacking multiple N^3^-probes in z-direction or twisting/rolling single probes makes them useful for advanced 3D neuronal measurements and peripheral nerve measurements and stimulation. Optical transparency and physical mesh structure allows to further increase the potential application range of the N^3^-MEAs toward three-dimensional cell culture. In the latter case, the N^3^-probe can be immersed into hydrogel-based solution before the solidification, allowing neuronal cells afterwards to grow freely through the culture and through the probe, and allowing a real three-dimensional electrophysiological recording from these 3D cell cultures without disturbance (see Figure S12).

## Ethics Statement

The experiments are done with the approval of the Landesumweltamt für Natur, Umwelt und Verbraucherschutz Nordrhein-Westfalen, Recklinghausen, Germany, number 84-02.04.2015.A173.

## Author Contributions

DK and AO conceived the study and designed fabrication protocols. DK, VM, and KS fabricated the devices. DK and JS performed electrical impedance characterization of electrodes. DK performed heart tissue experiments. DK and VM performed retina experiments. All authors contributed to the writing and agreed to the submission of the manuscript.

### Conflict of Interest Statement

The authors declare that the research was conducted in the absence of any commercial or financial relationships that could be construed as a potential conflict of interest.

## Supplementary Material

Supplementary Movies 1, 2 Supplementary movies provide visual content on probe's mechanical stability, and on the set-up and operation of the probes.
