# Supplementary material for: N3-MEA Probes: Scooping Neuronal Networks
Source: Front Neurosci. 2019 Apr 10;13:320. doi: 10.3389/fnins.2019.00320 (PMC6467947; doi:10.3389/fnins.2019.00320)
Supplement: Supplementary Movies 1, 2 — Supplementary movies provide visual content on probe's mechanical stability, and on the set-up and operation of the probes. [file Data_Sheet_1.PDF]

Supplementary Information

for

N^3-MEA probes: Scooping Neuronal Networks

Dmitry Kireev[1,2*], Viviana Rincon Montes[1], Jelena Stevanovic[1], Kagithiri Srikantharajah[1], Andreas Offenhaeusser[1]

Negative results:

(1) The same probes were fabricated using thin SU-8 (SU-8 2002), spin-coated at 3000 rpm, resulting in 1.6 µm thickness from each side, and total of 3 µm. These SU-8 based probes are extremely unstable and get broken very easily as soon as suspended from the sacrificial substrate and dried out.

(2) The greatest failure of the work and on the probes of the current structure came from measurements of in vitro cellular activity of HL-1 cell line. As a main desire of the proposed probe was ability to culture hundreds *in vitro* cultures separately and simply record them with a single probe, we tried to perform such proof of principle experiments. The HL-1 cells were cultured on glass slides and PDMS-covered glass slides and were visibly active (beating and providing spontaneous action potentials). Nonetheless, we were not able to bring the probe in a stable connection to the *in vitro* cells, i.e. we could not reach good sealing, therefore could not record APs. We believe this is related to the probe's relative mechanical mismatch and believe this can be solved by further decreasing the thickness of the probes, consequently improving the probe-tissue conformability. Another possible way to improve the sealing between the probe and tissue/cells is to prolong the contact time towards hours (rather than minutes as in our experiments). This will, however require multiple changes to the set-up, as it will require in-incubator recordings. The proposed method is schematically shown in the Supplementary Figure S9. In short, the probe can be fixed to a flexi-connector instead of the PCB board, and semi-permanently fixed to a Petri dish. Then, a sort of hydrogel-based neuronal culture can be seeded and grown through the mesh of the probe. Once the culture is ready, the compound can be taken out of the incubator and measured without disturbing the probe-cell interface.

(3) The first extracellular recordings from 3D- hydrogel-based neuronal assembly turned out to be unsuccessful as well. The main reason is an improper handling of the 3D organelles, as it was handled similarly to the heart tissue (that is more robust). We believe there is a way to measure

the neurons grown in 3D manner by the means of the NNN-probes in a non-invasive manner. The proposed method is schematically will be shown in the new Supplementary Figure S10.

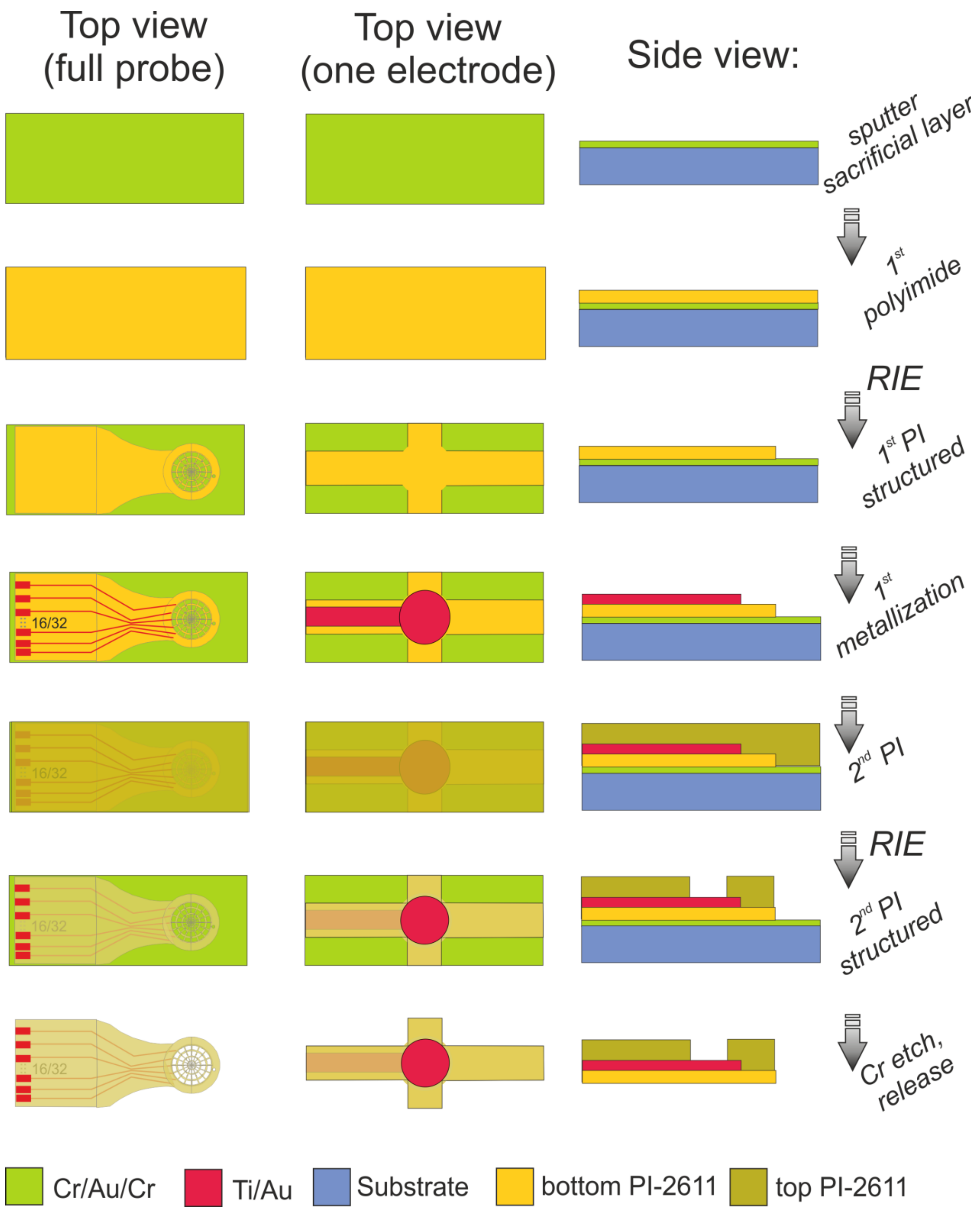

Figure S1. Overview of the N^3-MEA fabrication steps with three different views: top view on the whole probe; top view on one electrode; side view on one electrode.

This is a provisional file, not the final typeset article

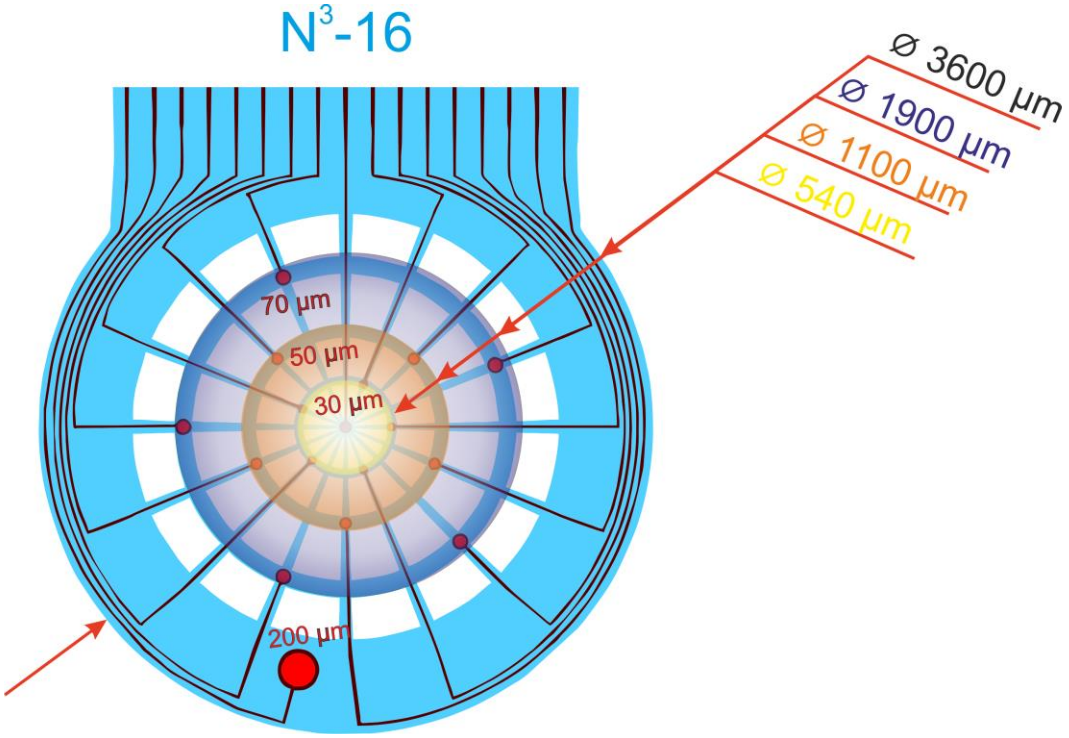

Figure S2. Sketch of the middle array of the N^3-16 probe with shown diameters of the electrodes (30 µm in the inner circled area, 50 µm in the middle area, 70 µm in the outer area) as well as dimensions of the whole chip.

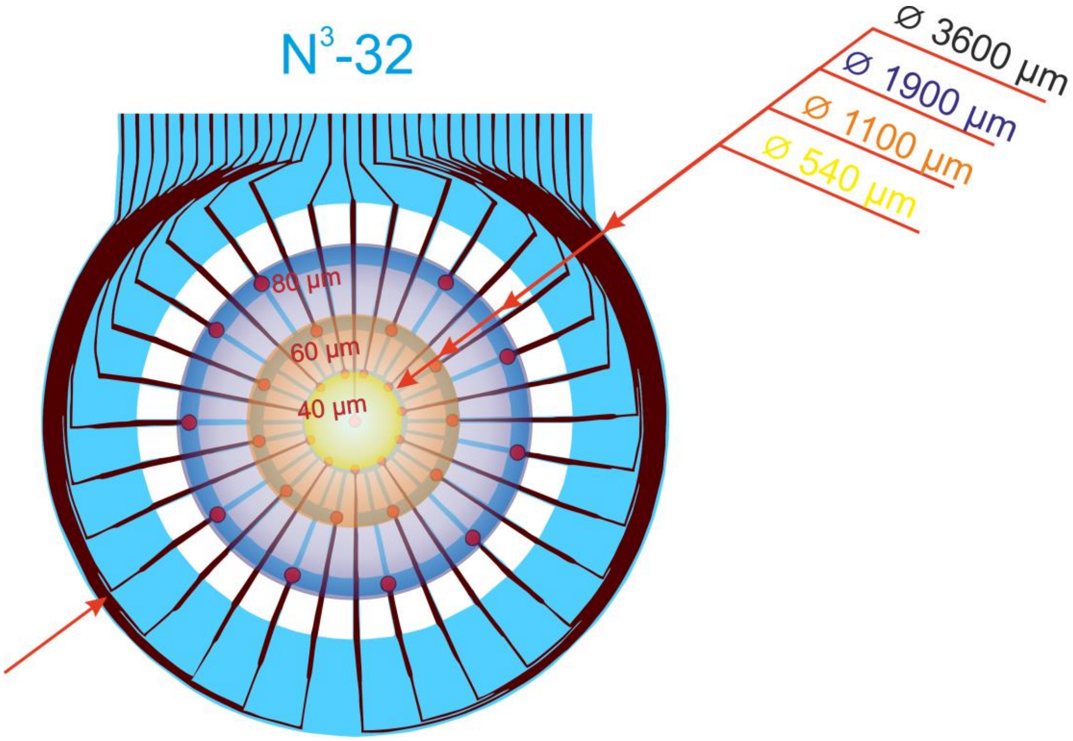

Figure S3. Sketch of the middle array of the N^3-32 probe with shown diameters of the electrodes (40 µm in the inner circled area, 60 µm in the middle area, and 80 µm in the outer area) as well as dimensions of the whole chip.

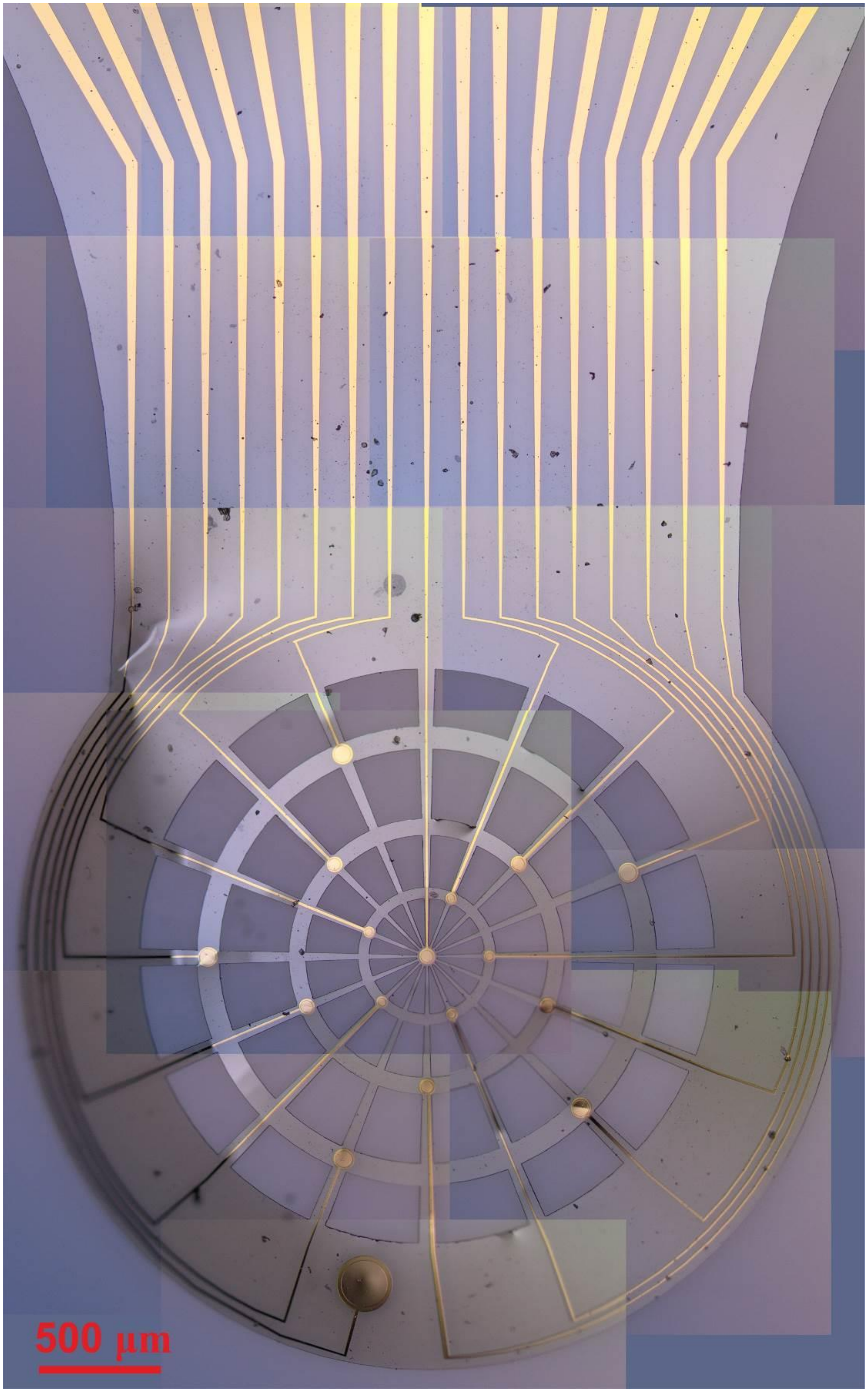

49 **Figure S4**. A panoramic assembly of a set of optical pictures of the N^3-16 probe.

This is a provisional file, not the final typeset article

50 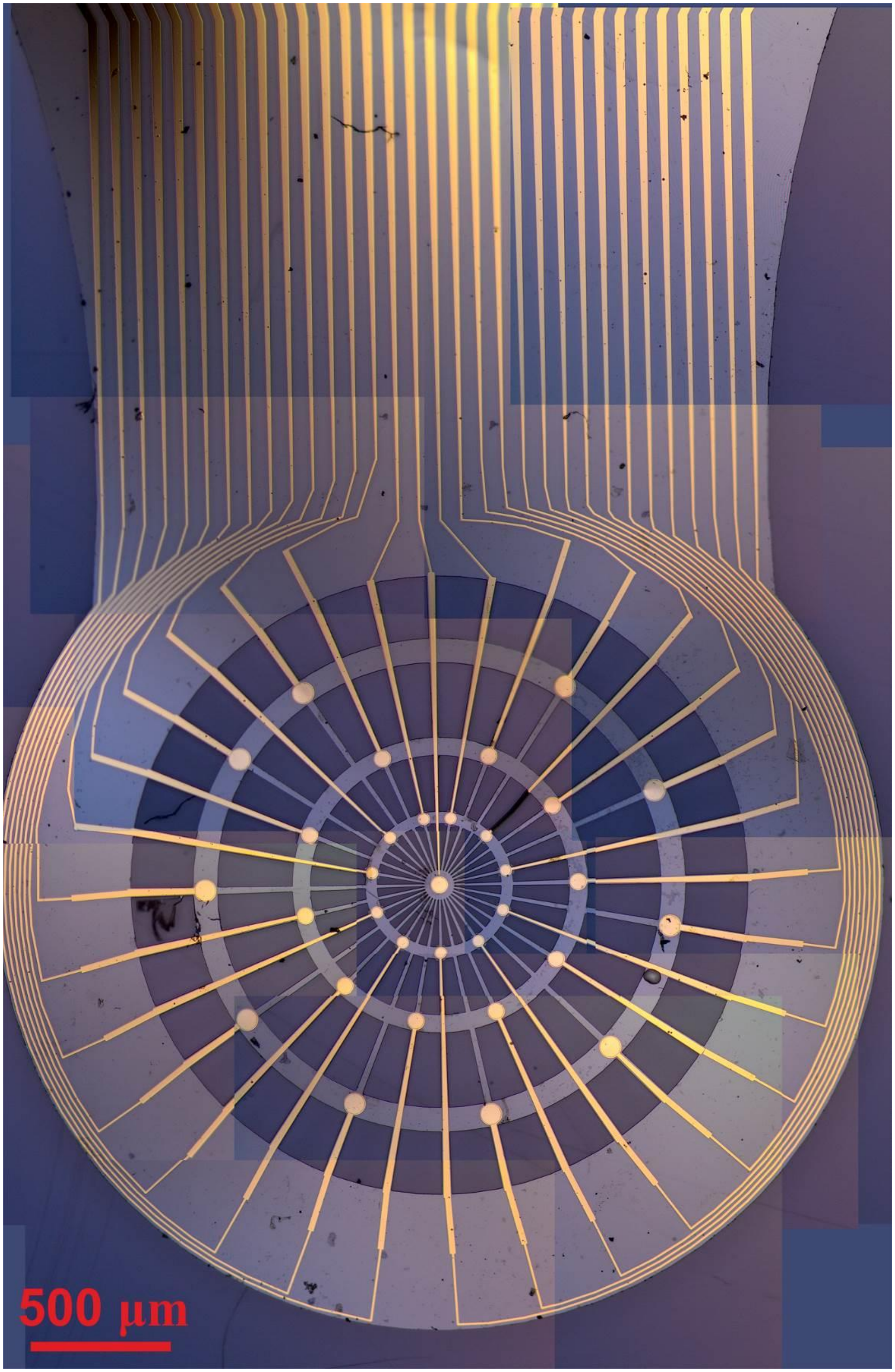

51 **Figure S5**. A panoramic assembly of a set of optical pictures of the N^3-32probe.

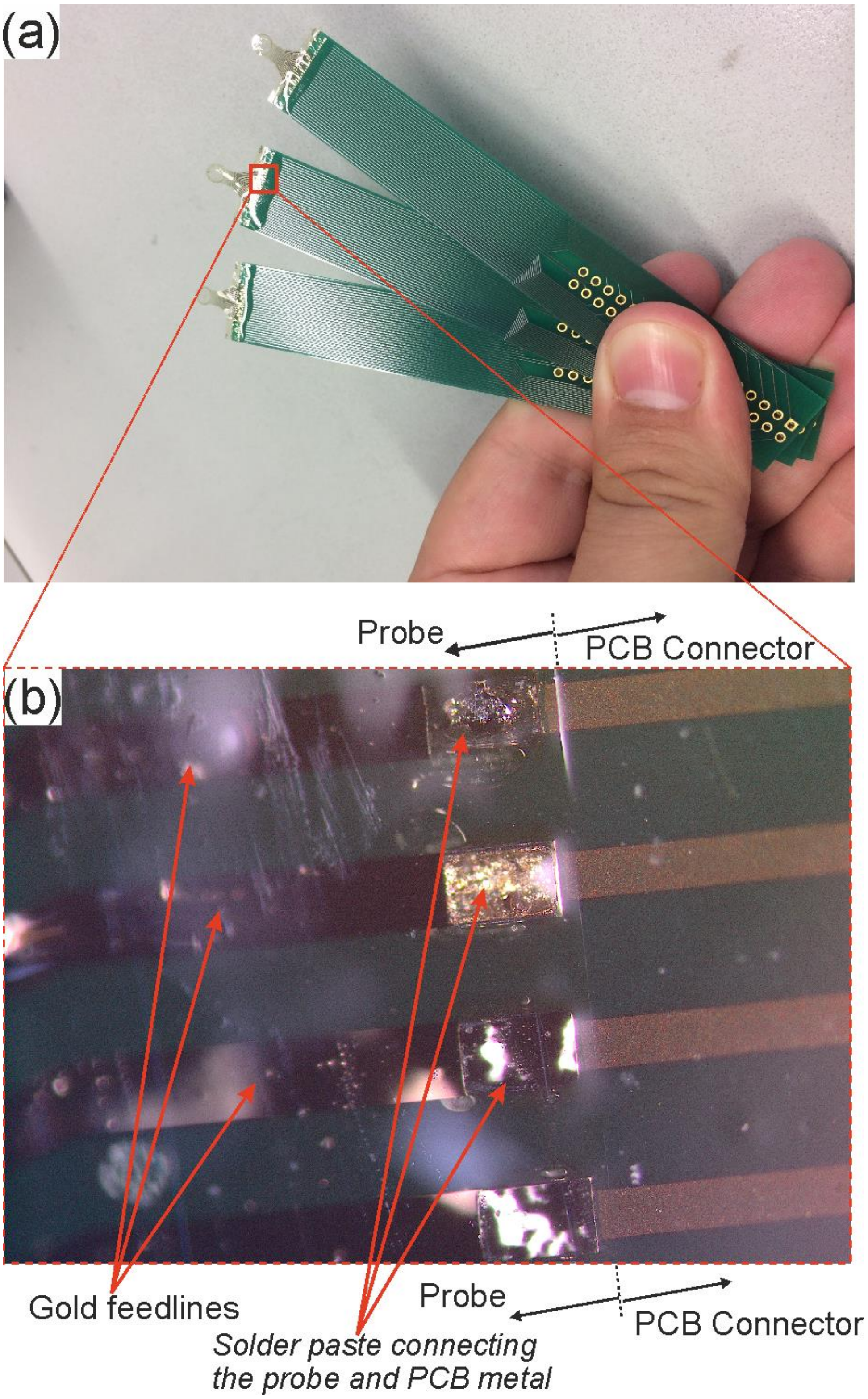

Figure S6. (a) Pictures of the N^3-16 and N^3-32 MEAs after bonding to a PCB carrier boards. (b) Close-up optical images of the probe-PCB connection. The Probe is on the left and is flip-chip bonded onto the PCB board using a special solder paste (see materials and methods section). The rather uneven lightning and contrast of the image is caused by the epoxy passivation on top.

This is a provisional file, not the final typeset article

Table S1. Values of impedance at 1kHz of one probe before and after a series of electrophysiological measurements. It seems that two electrodes got damaged during the measurements, #2 and #31, making in 94% of electrodes in a perfectly working condition. One electrode, #1 has improved in impedance, which might have been a result of an incorrect initial measurements, therefore was also taken out of the final calculation of the impedance change. As calculated from the 29 other electrodes, the impedance is increased in 32±11%.

# ELECTRODE	Impedance @ 1KHz, Ohm		ΔZ/Z, %	Electrode diameter, μm
	BEFORE	AFTER		
**1**	2.57E+05	1.82E+05	-29%	60
**2**	1.45E+05	4.07E+06	2718%	80
**3**	5.01E+05	6.46E+05	29%	40
**4**	2.57E+05	3.31E+05	29%	60
**5**	1.58E+05	2.24E+05	41%	80
**6**	5.75E+05	6.61E+05	15%	40
**7**	2.75E+05	3.24E+05	17%	60
**8**	1.66E+05	2.09E+05	26%	80
**9**	6.03E+05	6.92E+05	15%	40
**10**	2.88E+05	3.80E+05	32%	60
**11**	1.70E+05	2.29E+05	35%	80
**12**	5.75E+05	7.41E+05	29%	40
**13**	1.82E+05	2.14E+05	17%	80
**14**	2.82E+05	3.80E+05	35%	60
**15**	5.62E+05	7.76E+05	38%	40
**16**	2.75E+05	3.98E+05	45%	60
**17**	5.50E+05	7.24E+05	32%	40
**18**	2.88E+05	4.17E+05	45%	60
**19**	1.62E+05	2.24E+05	38%	80
**20**	6.03E+05	8.51E+05	41%	40
**21**	2.82E+05	4.47E+05	58%	60
**22**	1.62E+05	2.29E+05	41%	80
**23**	6.17E+05	7.94E+05	29%	40
**24**	2.88E+05	3.98E+05	38%	60
**25**	1.51E+05	2.24E+05	48%	80
**26**	5.01E+05	6.92E+05	38%	40
**27**	6.31E+06	6.92E+06	10%	60
**28**	1.66E+05	2.09E+05	26%	80
**29**	5.50E+05	7.24E+05	32%	40
**30**	2.63E+05	3.47E+05	32%	60
**31**	1.70E+05	4.57E+06	2592%	80
**32**	5.89E+05	7.76E+05	32%	40

# ELECTRODE	Impedance @ 1KHz		ΔZ/Z, %
	BEFORE	AFTER	
**3**	5.01E+05	6.46E+05	29%
**4**	2.57E+05	3.31E+05	29%
**5**	1.58E+05	2.24E+05	41%
**6**	5.75E+05	6.61E+05	15%
**7**	2.75E+05	3.24E+05	17%
**8**	1.66E+05	2.09E+05	26%
**9**	6.03E+05	6.92E+05	15%
**10**	2.88E+05	3.80E+05	32%
**11**	1.70E+05	2.29E+05	35%
**12**	5.75E+05	7.41E+05	29%
**13**	1.82E+05	2.14E+05	17%
**14**	2.82E+05	3.80E+05	35%
**15**	5.62E+05	7.76E+05	38%
**16**	2.75E+05	3.98E+05	45%
**17**	5.50E+05	7.24E+05	32%
**18**	2.88E+05	4.17E+05	45%
**19**	1.62E+05	2.24E+05	38%
**20**	6.03E+05	8.51E+05	41%
**21**	2.82E+05	4.47E+05	58%
**22**	1.62E+05	2.29E+05	41%
**23**	6.17E+05	7.94E+05	29%
**24**	2.88E+05	3.98E+05	38%
**25**	1.51E+05	2.24E+05	48%
**26**	5.01E+05	6.92E+05	38%
**27**	6.31E+06	6.92E+06	10%
**28**	1.66E+05	2.09E+05	26%
**29**	5.50E+05	7.24E+05	32%
**30**	2.63E+05	3.47E+05	32%
**32**	5.89E+05	7.76E+05	32%
		Mean:	***32%***
		SD:	***11%***

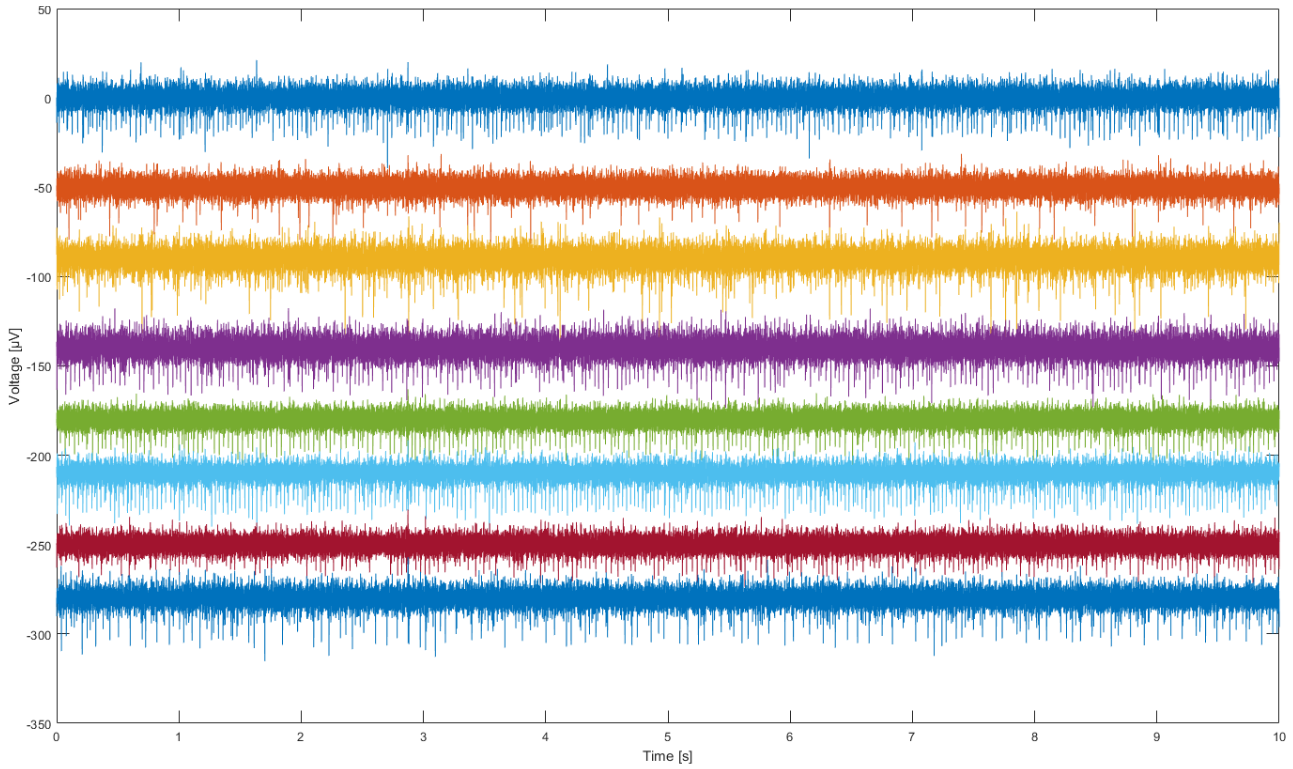

Figure S7. Spontaneous activity of retinal ganglion cells captured in eight different channels recorded from another piece of retina (different to the one in main manuscript).

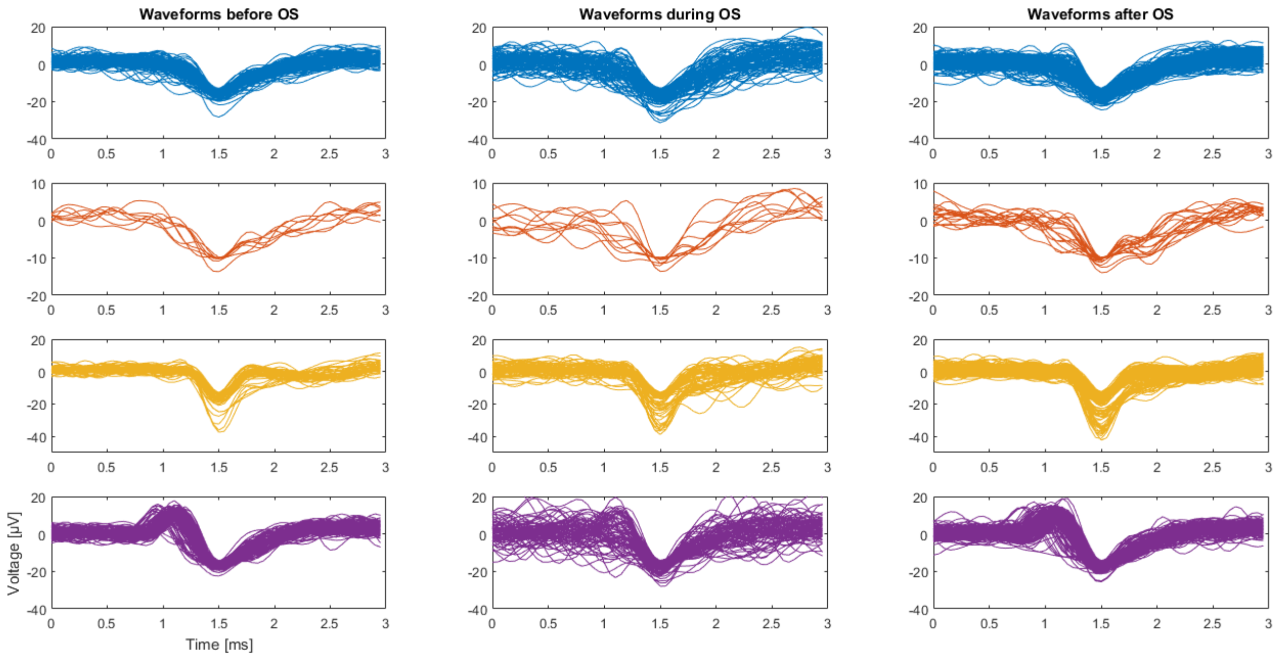

Figure S8. Waveforms of retinal spikes before, during, and after optical stimulation (OS). Each row correspond to the waveforms of the recordings shown in Figure 6 in the manuscript (same color code). Here, the presence of more than one spiking unit among the recording channels and within a same electrode can be observed, as different spike shapes and amplitudes are detected.

This is a provisional file, not the final typeset article

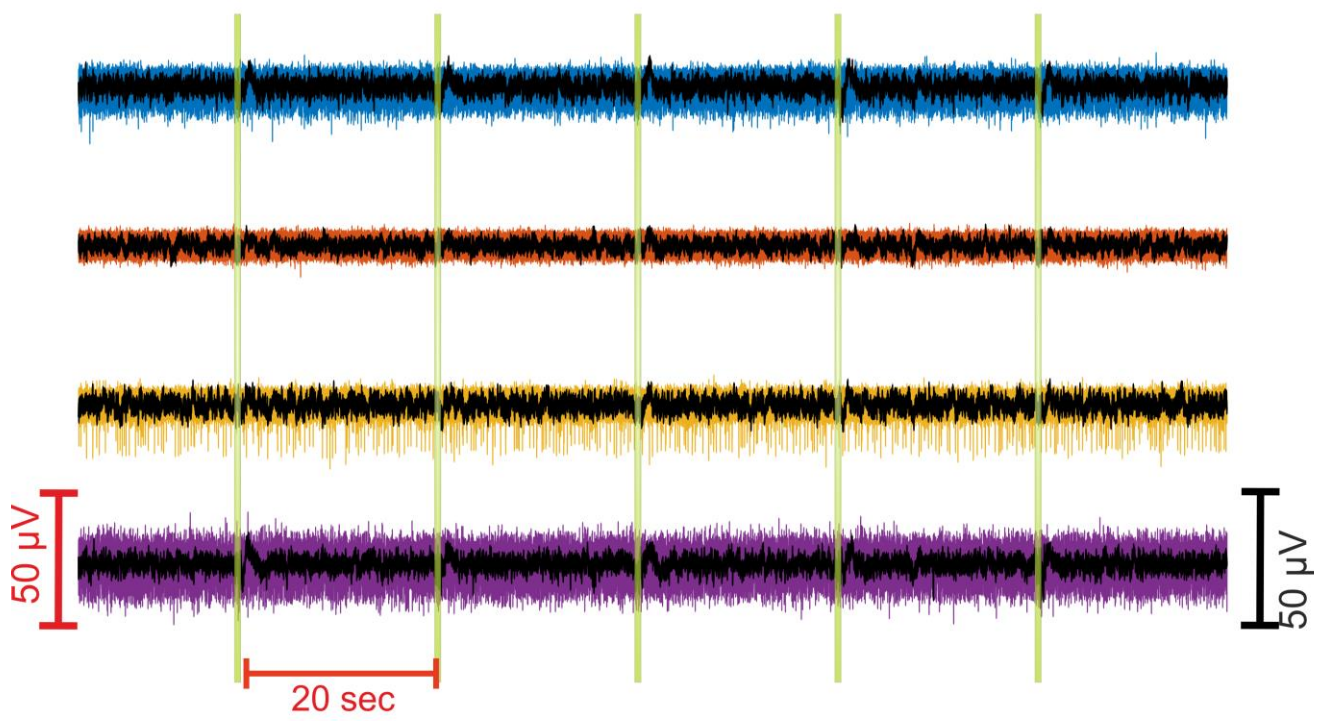

Figure S9. Optical stimulation to hypoxic/ischemic retina. The spiking activity (color coded according to Figure 6 in the manuscript) and low frequency signals – LFPs – (in bold black) captured by four electrodes from an explanted retina after one hour and 20 minutes without fresh oxygenated medium. Optical stimulation with a 500 ms light pulse (green highlights) every 20 seconds was carried out to confirm light evoked responses.

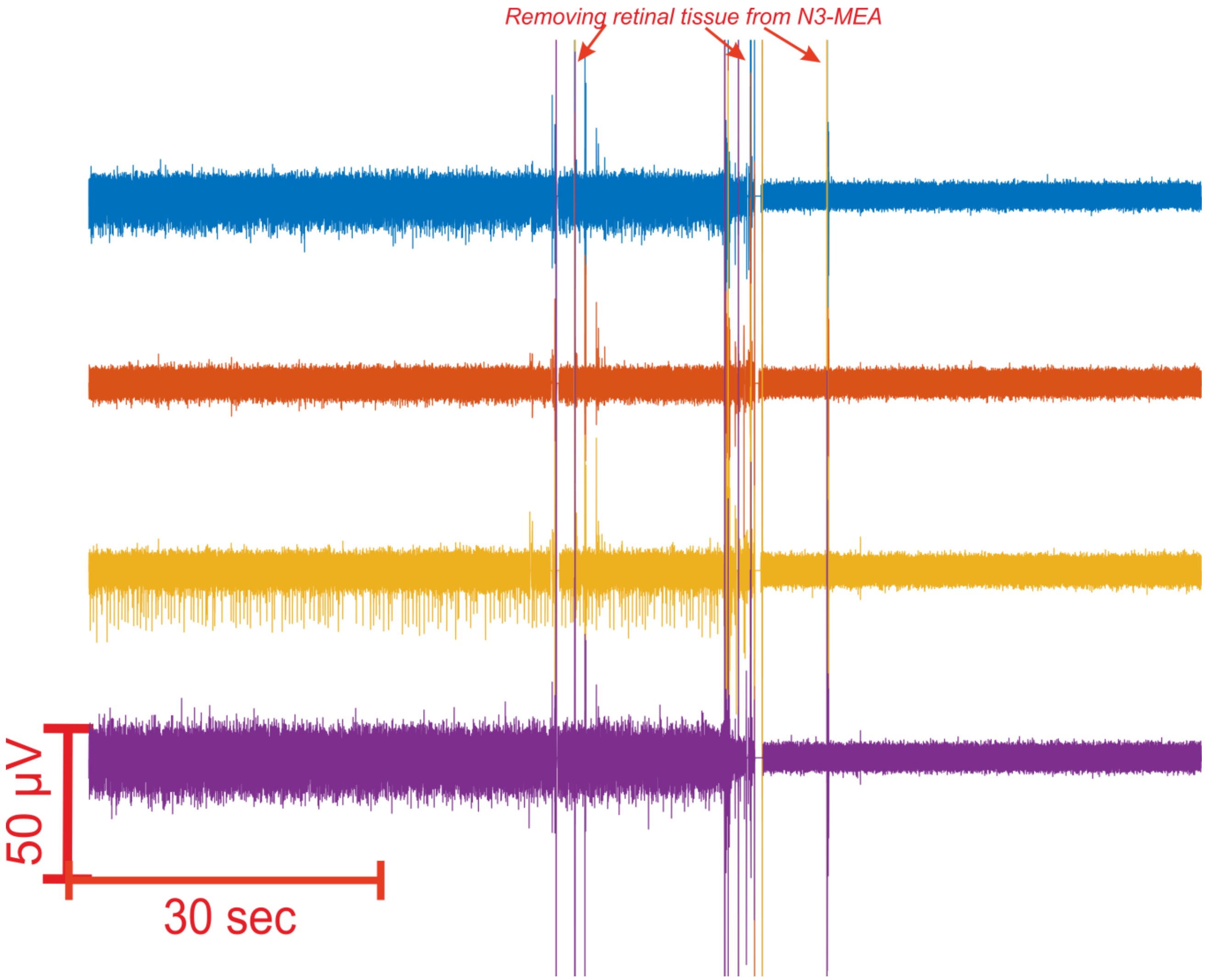

Figure S10. Electrical activity during tissue removal from N^3 probe. The electrical activity (color coded according to Figure 4 in the manuscript) of four channels is exhibited while the retinal tissue, after one hour and 20 minutes of a hypoxic/ischemic state, was removed from the chip. Removal of the tissue is seen in the electrical recordings as high amplitude peak artifacts. After the tissue was completely removed, the spiking activity observed at the yellow channel was completely gone, confirming that the origin of the spiking activity was coming from the retinal tissue. In the other three channels, the background noise was reduced when the tissue was removed, as the electrical resistance of the tissue was not present anymore.

This is a provisional file, not the final typeset article

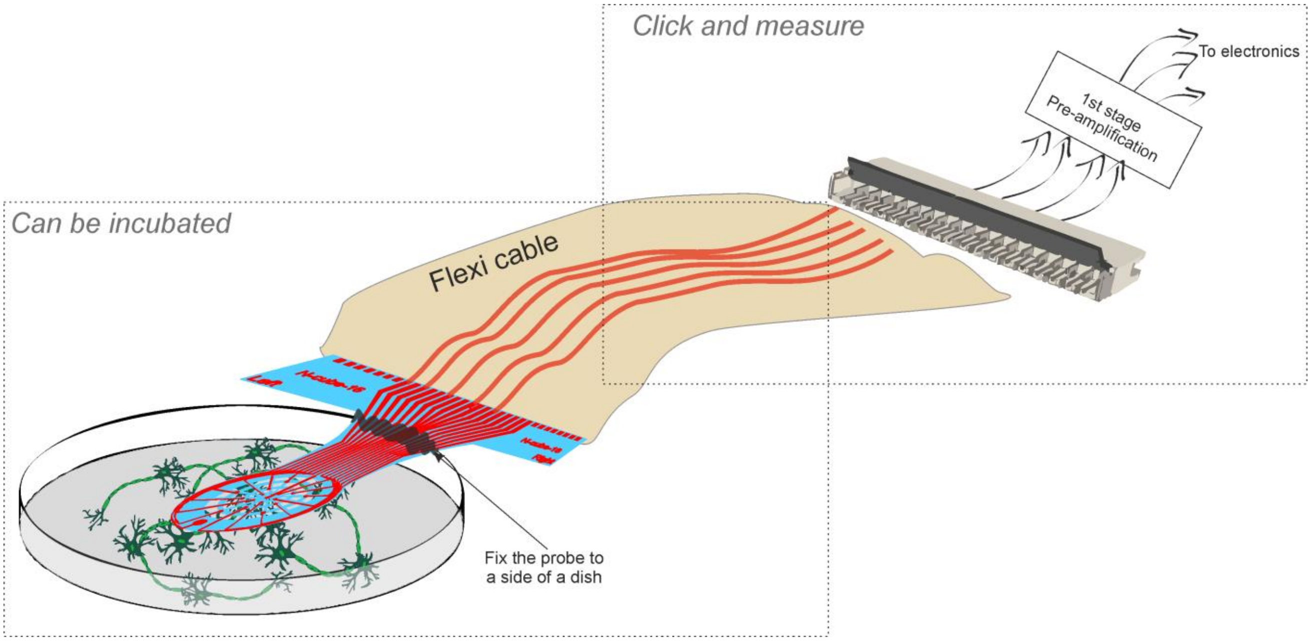

Figure S11. Proposed schematic of using the N3-probes for longer incubation with neuronal or other in vitro cell cultures. Longer incubation does not allow standard electronics to be inserted directly into the incubator, therefore we propose to (i) bond the N3-prove to a flexi connector (that can be incubated), (ii) fix the probe/flexi connector to a side of a petri dish allowing the whole assembly to do into incubator and outside, further connect with a simple plug-and measure kind of connector (e.g. ZIF) and perform electrical measurements without disturbing the probe/cells interface.

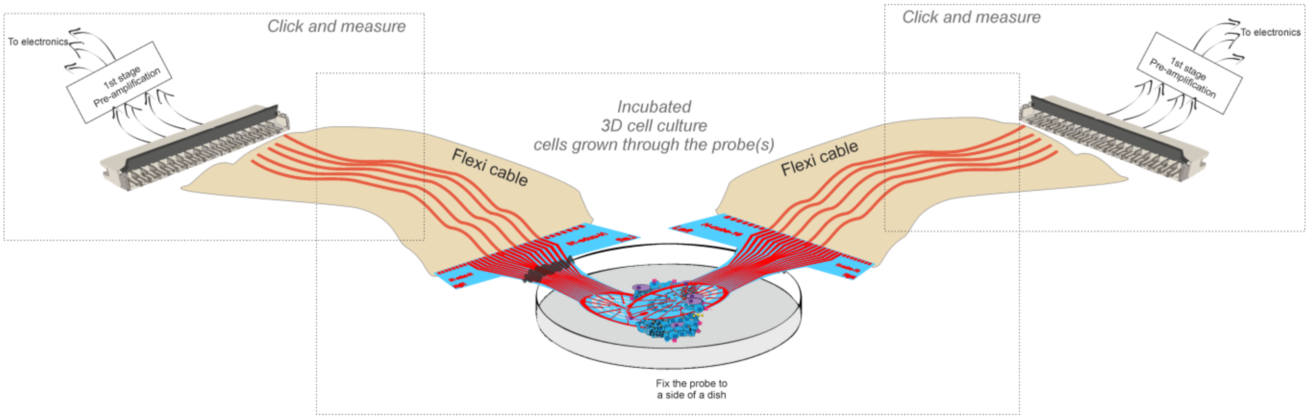

Figure S12. A more complex schematic proposed, where a 3D neuronal cel culture can be measured by using multiple probes fixed to a dish on top of each other, and growing the hydrogel-supported neurons through the probes.
